# Single-nucleus profiling of postmortem diffuse midline gliomas identifies mitochondrial biogenesis as a resistance mechanism to imipridone therapy

**DOI:** 10.1093/neuonc/noag119

**Published:** 2026-05-24

**Authors:** Masahiro Okada, Bavani Subramaniam, Baobao Geng, Jangham Jung, Bohyeon Yu, Felicia Lin, Yu Fu, Yishu Wang, Weiqiang Yu, Sandra Laternser, Nalin Gupta, Sabine Müller, Lin Wang, Aaron Diaz, Javad Nazarian

**Affiliations:** Department of Neurological Surgery, University of California, San Francisco, California, USA; Center for Cancer and Immunology Research, Children’s National Hospital, Washington, District of Columbia, USA; State Key Laboratory of Common Mechanism Research for Major Diseases, Suzhou Institute of Systems Medicine, Chinese Academy of Medical Sciences & Peking Union Medical College, Suzhou, Jiangsu, China; Department of Neurological Surgery, University of California, San Francisco, California, USA; Department of Neurological Surgery, University of California, San Francisco, California, USA; Department of Neurological Surgery, University of California, San Francisco, California, USA; State Key Laboratory of Common Mechanism Research for Major Diseases, Suzhou Institute of Systems Medicine, Chinese Academy of Medical Sciences & Peking Union Medical College, Suzhou, Jiangsu, China; State Key Laboratory of Common Mechanism Research for Major Diseases, Suzhou Institute of Systems Medicine, Chinese Academy of Medical Sciences & Peking Union Medical College, Suzhou, Jiangsu, China; State Key Laboratory of Common Mechanism Research for Major Diseases, Suzhou Institute of Systems Medicine, Chinese Academy of Medical Sciences & Peking Union Medical College, Suzhou, Jiangsu, China; Children’s Research Center, University Children’s Hospital Zurich, University of Zurich, Zurich, Switzerland; Department of Neurological Surgery, University of California, San Francisco, California, USA; Department of Neurological Surgery, University of California, San Francisco, California, USA; Department of Neurology, University of California, San Francisco, California, USA; State Key Laboratory of Common Mechanism Research for Major Diseases, Suzhou Institute of Systems Medicine, Chinese Academy of Medical Sciences & Peking Union Medical College, Suzhou, Jiangsu, China; Department of Neurological Surgery, University of California, San Francisco, California, USA; Children’s Research Center, University Children’s Hospital Zurich, University of Zurich, Zurich, Switzerland

**Keywords:** biomarkers, diffuse intrinsic pontine glioma, diffuse midline glioma, DIPG, DMG, Dordaviprone, imipridone, metabolism, Modeyso, ONC201, ONC206, scATAC-seq, scRNA-seq, single-cell genomics, TME, tumor microenvironment

## Abstract

**Background:**

Imipridone ONC201 is the first FDA-approved therapy for H3K27-altered diffuse midline glioma; however, clinical responses remain limited. Defining tumor-intrinsic determinants and microenvironmental, extrinsic factors that shape sensitivity or resistance to imipridones will identify actionable therapeutic opportunities and inform improved clinical strategies.

**Methods:**

To identify mechanisms of imipridone resistance, we obtained postmortem brain tissue from DMG patients who had received imipridones and/or standard care. Single-nucleus RNA and open-chromatin sequencing were performed on *N* = 22 cases. Immunofluorescence-based myeloid phenotyping was performed on *N* = 46 cases. Mitochondrial copy-number analysis was performed on *N* = 19 cases. Validation of imipridone sensitivity, its effect on mitochondrial density, and its synergy with inhibition of mitochondrial biogenesis were assessed in DMG primary cells.

**Results:**

We established a single-cell RNA/open-chromatin atlas from postmortem DMG cases and found imipridone treatment resulting in regressed mesenchymal transition, reduced myeloid-derived suppressive cells, and reversed aberrant H3K27-altered enhancer activity. Resistant tumors showed increased mitochondrial density, turnover, and membrane potential. Mitochondrial biogenesis and PPARGC1A emerged as resistance biomarkers and actionable targets.

**Conclusions:**

These studies implicate mitochondrial biogenesis as a biomarker of imipridone resistance and a focus for the development of combinatorial strategies to provide effective therapeutic options for a challenging pediatric brain tumor.

Key Points

- We identify mitochondrial biogenesis and PPARGC1A as biomarkers of resistance to imipridone therapy and as therapeutic targets.
- We present an atlas of single-cell RNA and open-chromatin data from *N* = 22 postmortem specimens of diffuse midline glioma.

Importance of the Study

We identify mitochondrial biogenesis and *PPARGC1A* as biomarkers of resistance to imipridone therapy and as therapeutic targets for imipridone adjuvant therapy. Imipridone therapy regresses mesenchymal transition and myeloid-derived suppressive cells and thus may synergize with emerging immunotherapies. We present an atlas of single-cell RNA and open-chromatin data from *N* = 22 postmortem diffuse midline glioma specimens.

Diffuse midline glioma, H3K27-altered (DMG), is a malignant pediatric brain cancer with a dismal median overall survival of 9-11 months from diagnosis.^1^^,^^2^ Significant inter-patient heterogeneity in DMG cellular composition has been reported,^3^ indicating that patient age and local microenvironment shape the balance between DMG cells with an oligodendrocyte progenitor cell (OPC) phenotype and those with a mesenchymal (MES) phenotype. The MES phenotype is associated with resistance to ionizing radiation (IR).^4^^,^^5^

Imipridones are orally available, blood-brain barrier (BBB) penetrant drugs that have been reported to increase DMG median overall survival in clinical trials.^6-9^ The imipridone Dordaviprone was approved by the Food and Drug Administration (FDA) as the first systemic therapy for patients with H3K27M-mutant DMG in August 2025.^10^ Several clinical trials are actively investigating the drug’s efficacy as a single agent or in combination with other treatments, including PNOC0022 (NCT05009992) and a placebo-controlled phase III clinical trial (NCT05580562). Despite its early clinical promise,^9^ imipridones show variable efficacy across the DMG patient population, highlighting the need to identify mechanisms of response and resistance to inform rational combination strategies.

To address these gaps, we profiled *N* = 22 postmortem specimens from human DMGs treated with the imipridone ONC201 or standard care (surgical resection and IR) using single-nucleus RNA sequencing (snRNA-seq) and single-cell assay for transposase-accessible chromatin by sequencing (scATAC-seq). This cohort included imipridone and standard-care-treated tumors from both the pons and thalamus. We and others have shown that imipridones impair mitochondrial function by activating the proteolytic activity of caseinolytic mitochondrial matrix peptidase proteolytic subunit (ClpP).^9^^,^^11-13^ This metabolic perturbation results in increased H3K27 trimethylation, which is hypothesized to partially reverse the pathology of H3K27M mutations that define DMG.^9^ Here, we report that this phenomenon also occurs in the clinical setting. Our data show that imipridone treatment correlates with regression of MES transition and a decrease in myeloid-derived suppressive cells. Our study indicates that imipridone therapy may synergize with emerging immunotherapies for DMGs. Single-cell analysis of clinical specimens and in vitro functional studies demonstrated that mitochondrial biogenesis is a resistance mechanism to imipridone therapy and a target for the development of combinatorial strategies.

## Methods

### Tumor Tissue Acquisition and Ethical Approval

All postmortem specimens were collected following written informed consent by the patient’s guardian, in accordance with the protocols approved by the Institutional Review Board of Children’s National Hospital (Pro00001339, Pro00000747, Pro00000463, Pro00000747) and the Swiss Ethics Committee (BASEC Nr. 2019-00615 and 2024-00086) in Switzerland. Tissue samples were processed within 3-70 hours after autopsy, depending on the location of sample collection and other logistical factors. Tumor specimens were confirmed by pathological staining and analysis performed by the Department of Pathology at Children’s National Hospital. Tissue samples were preserved through flash freezing in liquid nitrogen, fixation in 10% neutral buffered formalin, or cryopreservation in cell freezing media supplemented with DMSO. All samples were de-identified and assigned unique numerical identifiers for storage at Children’s National Hospital. De-identified flash-frozen samples were then provided to UCSF for further analysis, with approval from the UCSF Institutional Review Board. All experiments were carried out in conformity with the principles set out in the WMA Declaration of Helsinki and the Department of Health and Human Services Belmont Report. All patients provided informed written consent.

### Statistics and Reproducibility

No statistical method was used to predetermine the sample size of the clinical cohort profiled. All available postmortem DMG specimens were included. No data were excluded from the analyses. The MAST^14^ two-sided likelihood-ratio test between hurdle models was used to determine differential gene expression between single-cell datasets, and *P*-value were adjusted for multiple hypothesis testing via Storey’s method.^15^ Tests for differences in snRNA-seq-derived cell-type proportions were performed via the propeller method.^16^ Fisher’s exact test was used to test for genotype-phenotype associations. An unpaired two-sided t-test was used to assess differences in qPCR and cell proliferation assays.

### Nuclei Isolation from Frozen Tissue

For snRNA-seq, nuclei were extracted from frozen tissues following the ‘Van Helsing’ protocol developed by L. Martelotto, Melbourne, Centre for Cancer Research, Victorian Comprehensive Cancer Centre and available from 10x Genomics (dx.doi.org/10.17504/protocols.io.bdeni3de). Briefly, frozen tissues were digested mechanically in a Dounce grinder with 500 µL of lysis buffer (Sigma). The lysate was strained through a 30-μm strainer, pelleted, washed, and resuspended in 500 µL PBS supplemented with 1% BSA and 0.2 U/µL RNase Inhibitor. Nuclei were stained by Vybrant DyeCycle Ruby Stain (Invitrogen, V10273) and isolated via a SONY SH800 Cell Sorter or BD FACSAria Fusion. For snATAC-seq assays, nuclei were purified via digitonin-based washing and resuspended in tagmentation buffer (10x Genomics, 2000193).

### 10x Genomics-Based scRNA-Seq/snRNA-Seq/snATAC-Seq

Single-cell/nucleus capture, reverse transcription, cell lysis, and library preparation for snRNA-seq were performed via the 10x Genomics platform per the manufacturer’s protocol. Approximately 15,000 nuclei or 25,000 live cells were loaded per capture. Sequencing was performed on an Illumina NovaSeq with 10x Genomics’ recommended parameters.

### SnRNA-Seq Data Pre-Processing

The pre-processing of snRNA-Seq data was performed as described previously.^17^ We used CellRanger (version 7.2.0) for alignment and gene expression quantification, following the guidelines on the CellRanger website (https://support.10xgenomics.com/single-cell-gene-expression/software/pipelines/latest/advanced/references#premrna). We filtered cells with > 5% mitochondrial read counts and fewer than 200 expressed genes. DoubletFinder (version 2.0.2)^18^ was used to remove doublets, using the first 10 principal components and default parameters.

### Dimensionality Reduction, Clustering, and Cell-Type Classification for snRNA-Seq

The snRNA-seq data were processed with Seurat v5.^19^ Data were normalized via the LogNormalize method with scale.factor = 10,000 using the NormalizeData function. Highly variable genes were identified via Seurat using the mean.var.plot method with default parameters. Based on these genes, a PCA was performed, and the first 10 principal components were retained for clustering and visualization via tSNE. Neoplastic cells were separated from non-neoplastic cells based on the presence of CNVs. InferCNV was used for the CNV analysis to identify neoplastic cells and non-neoplastic cells. Unbiased clustering was performed using the “FindClusters” function with the Louvain algorithm and a resolution parameter of 0.5. Cluster-specific genes were identified via the FindAllMarkers function in Seurat V5 via a MAST test.

### SnATAC-Seq Data Processing and Analysis

The CellRanger ATAC software (version 2.1.0) was used for read alignment, deduplication, and identifying transposase cut sites (https://support.10xgenomics.com/single-cell-atac/software/pipelines/latest/algorithms/overview). The output matrix of CellRanger was further processed via the snapATAC package (https://github.com/r3fang/SnapATAC ).^20^ We selected the highest quality barcodes for each case based on two criteria: 1) number of filtered fragments > 1000; 2) fragments in promoter ratio (FRiP) > 0.2 for the case. Clustering was performed using Seurat V5 SNN-graph clustering via the “FindClusters” routine, with gene body-accessibility scores generated by the snapATAC package as input. Transcription-factor (TF) motif frequency deviations from a data-driven background model were calculated via the computeDeviations function in chromVAR (version 1.6.0) with default parameters,^21^ using only neoplastic cells as input. Differential motif deviances were computed via a T-test and controlled for multiple-hypothesis testing via fdrtool.^15^ Differentially accessible regions, peaks, and motif enrichments on differential peaks (relative to a genome-wide background) were computed using snapATAC’s “findDAR,” “runMACSForAll,” and “runHomer,” respectively, run with default parameters. Here, the gene activity of cells generated by snapATAC (version 2.0) was used as input to perform CNV analysis. The bam file from 10x snATAC-seq data generated with CellRanger16 was used to perform SNV calling by pooling reads by patient and running the GATK best-practices pipeline. Variant assignments in single cells were then assessed via the VarTrix (version 1.0) tool.^22^ Variants were annotated with the Annovar software package.^23^

### Cell-Cell Communication Analysis

The CellChat package was used to assess cell-cell communication via interaction-network analysis.^24^ A Seurat object was used as input to CellChat, following their standard protocol as described at https://github.com/sqjin/CellChat. Circle and dot plots were generated using netVisual_aggregate and vertex.size = groupSize and netAnalysis_dot resp. Data from ONC201-treated, standard, and untreated cases were processed separately.

### Monocytic Cell Classification and Analysis

To classify the activation status of monocytic-lineage cells, signature genes of classically (M1: *CCL2/3/4/5/8*, *CCR7*, *CD74*, *CSF2*, *CXCL10*, *HLA-DRA*/*B*, *IFNG*, *IL1B*, *IL1R1*, *IL6*, *INOS*, *IRF5*, *NFKB1*, *TLR2*/*4*, *TNF*) and alternatively activated (M2: *ARG1*, *CD74*, *CCL1/17/22/*, *CXCL16*, *CXCR4*, *HLA-DRA*/*B*, *IL10*, *IL4*, *IRF4*, *MRC1*, *NFKB1*, *TGFB1*, *TNF*) phenotypes were aggregated from prior reports.^25-29^ The M1 and M2 score for each cell was calculated via AddModuleScore function with the above M1/M2 signatures as input. This routine compares average signature levels to a data-driven background distribution. Cells were assigned M0 status if the M1 and M2 signature scores were both less than 0.0. Otherwise, activation status was determined by the higher of the M1 and M2 signature scores.

### Cell Culture

SF8628 cells were maintained in DMEM (high glucose) supplemented with 10% FBS (Gibco), 1% 5000 U/mL penicillin and 5000 µg/mL streptomycin solution (Gibco), and 1% GlutaMAX-I Supplement (Gibco). DMG cell lines (CNHDMG760, SF10693, HSJDDIPG007, and KISPIDMG105) were maintained in 1:1 solution of DMEM/F12 (Invitrogen) and Neurobasal-A Medium (Invitrogen) supplemented with 1% HEPES Buffer Solution (1M), 1% MEM Sodium Pyruvate Solution (100mM), 1% MEM Non-Essential Amino Acids (10mM), 1% GlutaMAX-I Supplement, 1% 5000 U/mL penicillin and 5000 µg/mL streptomycin solution, 1xB-27 Supplement (-Vitamin A) (Invitrogen), 20 ng/mL human-EGF (Peprotech), 20 ng/mL human-bFGF (Peprotech), 10 ng/mL human-PDGF-AA (Peprotech), 10 ng/mL human-PDGF-BB (Peprotech), and 0.1% heparin solution (0.2%) (StemCell Technologies, Inc.). Clinical grade ONC201 was provided by Chimerix.(Jazz Pharmaceuticals). SR-18292 was purchased from Selleck S8528. Drug sensitivity was determined via the CellTiterGlo Luminescent Cell Viability Assay Kit (Promega) 96 h after treatment. Dose responses were calculated via the R package drc, using a 2-parameter log-logistic model. SynergyFinder was used for calculating drugs combination synergistic effect.^30^

### RNA Extraction and qPCR

DMG cells were harvested after ONC201 treatment for 72 h and lysed by RLT Plus Buffer supplemented with 1% 2ME. Total RNA was extracted by using RNeasy Plus Mini Kit (QIAGEN), and 350 ng-1000 ng RNA were reverse transcribed by using PrimeScript IV 1st strand cDNA Synthesis Mix (TAKARA) as follows: 42°C for 20 min and 70°C for 15 min. 10 times diluted cDNA were subjected to gene expression by using Power Up SYBR Green Master Mix and 200 nM each primer. QuantStudio3 was used for measurement as follows: 50°C for 2 min, 95°C for 2 min and 40 cycles of 95°C for 15 sec and 60°C for 1 min. The following primers were used for amplification: HPRT1_For (CATTATGCTGAGGATTTGGAAAGG), HPRT1_Rev (CTTGAGCACACAGAGGGCTACA), PPARGC1A_For (CCAAAGGATGCGCTCTCGTTCA), and PPARGC1A_Rev (CGGTGTCTGTAGTGGCTTGACT). The ddCt method was used for comparison.

### Flow Cytometric Analysis of Mitochondria

DMG cells were harvested after ONC201 treatment and stained with 30 nM MitoTracker Green, 30 nM MitoTracker Deep Red (Cell Signaling), and 1/1000 FVD780 (Invitrogen) in MACS buffer at room temperature for 15 min. Data were acquired with a BD FACSAria Fusion and analyzed with FlowJo.

### TMA Sections

Tumor tissues were fixed, dehydrated, embedded in paraffin, and cut into 5 µm-thick sections. Tissue sections were stained using hematoxylin and eosin (H&E) and reviewed by a pathologist at the University Children’s Hospital Zurich to confirm the presence of tumor cells. For each patient, 2-4 distinct tissues were selected and punched to obtain *N* = 3 cores with diameters of 0.6 mm. The TMA was assembled using the TMA Grand Master Panoramic 250 Scanner 3dHistec, with a total of 918 punch cores across two TMA blocks. The TMA blocks were 17 x 23 mm each.

### Immunofluorescence Staining

TMA slides were deparaffinized and rehydrated. Heat-induced epitope retrieval was performed using citrate (pH 6) and Tris (pH 9). Following DAPI staining, sequential antibody staining was performed via the Leica Bond BOND-MAX auto-stainer for CD68 (Thermo Fisher MS-397-PABX), CD163 (BioRad MCA1853), and Iba1 (Wako Chemical 019-19741). Slides were imaged via a Cell DIVE system (Leica Microsystems, Issaquah, WA). Complete punch cores were imaged at 20x magnification and subjected to autofluorescence subtraction and registration via baseline DAPI. Images were analyzed using QuPath (v.0.3.2).

### DNA Isolation and qPCR

DNA from tumor tissues was isolated using the Qiagen QIAamp DNA Mini Kit and quantified via Qubit. QPCR was performed in triplicate using a QuantStudio 7 real-time PCR system (Thermo Fisher Scientific). Primers targeting MT-ND6 were used to quantify mitochondrial DNA, and primers targeting chr11: 2170993-2171170 were used to quantify nuclear DNA.

Primers used:

MT-ND6:

Forward: AGGATTGGTGCTGTGGGTGAAAGA

Reverse: ATAGGATCCTCCCGAATCAACCCT

chr11: 2170993-2171170

Forward: AGGGTATCTGGGCTCTGG

Reverse: GGCTGAAAAGCTCCCGATTAT

### Data and Code Availability

The study data are available and can be visualized on the UCSC Cell browser (https://cells-test.gi.ucsc.edu/? ds=brain-dmg-multiomics+snrna-seq-data). All the custom code used is available from GitHub at https://github.com/CancerGenomicsLab/DMG_ONC201. The raw sequenced reads are available from the European Genome-Phenome Archive (EGAS50000001447).

## Results

### A Single-Nucleus Atlas of DMG Postmortem Specimens

**Figure 1.**
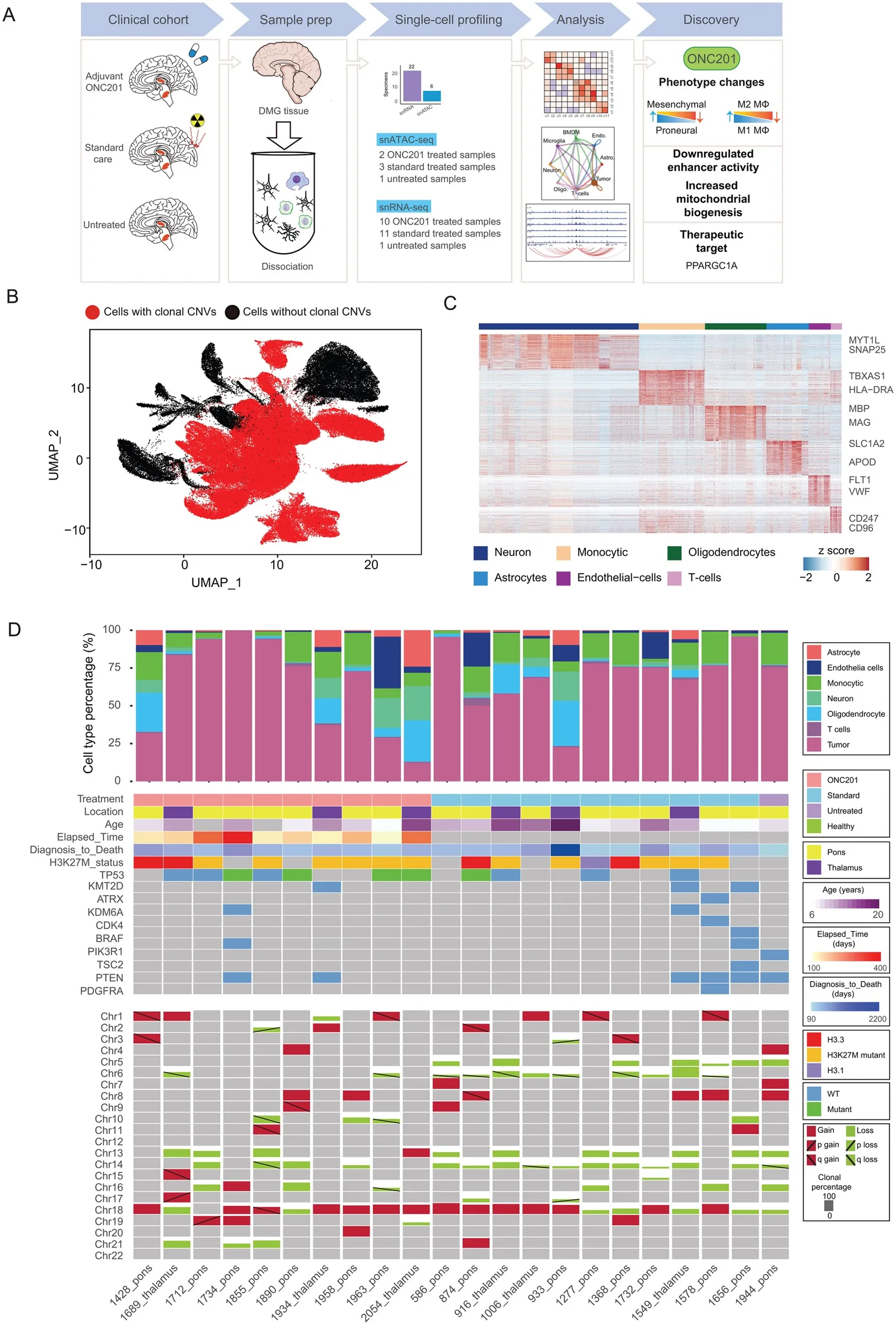
Single-cell genomics analysis of DMG postmortem samples from imipridone and standard-care-treated cases. (A) Overview of the study design. (B) UMAP plot of snRNA-seq data. Cells with CNVs are annotated. *N* = 22 tumor specimens were used. (C) Hierarchical clustering of cells without CNVs. Cluster-specific genes are highlighted. (D) Sample summary, including cellular composition, clinical data, copy-number variant, and single-nucleotide variant information. Created using images from SciDraw.io (Stoyo Karamihalev https://scidraw.io/drawing/395; John Chilton https://scidraw.io/drawing/221, 297, 350; Christophe Leterrier https://beta.scidraw.io/drawing/12), licensed under CC BY 4.0.

We profiled flash-frozen tumor specimens from DMG patients (*N* = 22), obtained at autopsy. Tumor burden was confirmed by a neuropathologist via H&E staining. All cases were validated as H3K27M-mutant via DNA sequencing and/or immunohistochemical staining. The DMG cohort included patients aged 4-20 years, with a male-to-female ratio of 55%. The clinical management of these cases included treatment with the imipridone ONC201 (*N* = 10), standard care/imipridone-naïve (*N* = 11), and one case was untreated (Figure 1A, Table S1). DMG specimens were subjected to single-nucleus RNA sequencing (snRNA-seq). We used our previously described pipelines^17^^,^^31-34^ for quality control, data preprocessing, and the separation of neoplastic cells from non-malignant neuroglia, immune cells, etc., based on expressed mutations (Figure 1B-D; Figures S1 and S2A-C). We found no correlation between the number of features detected and the time required for postmortem processing (Figure S1). The study data are available for visualization and interactive exploration via the UCSC Cell browser (https://cells-test.gi.ucsc.edu/?ds=brain-dmg-multiomics+snrna-seq-data).

### Single-Nucleus RNA Sequencing Demonstrates a Reduction in the Mesenchymal Phenotype and Myeloid-Derived Suppressive Cells in Imipridone-Treated Postmortem DMG Specimens

**Figure 2.**
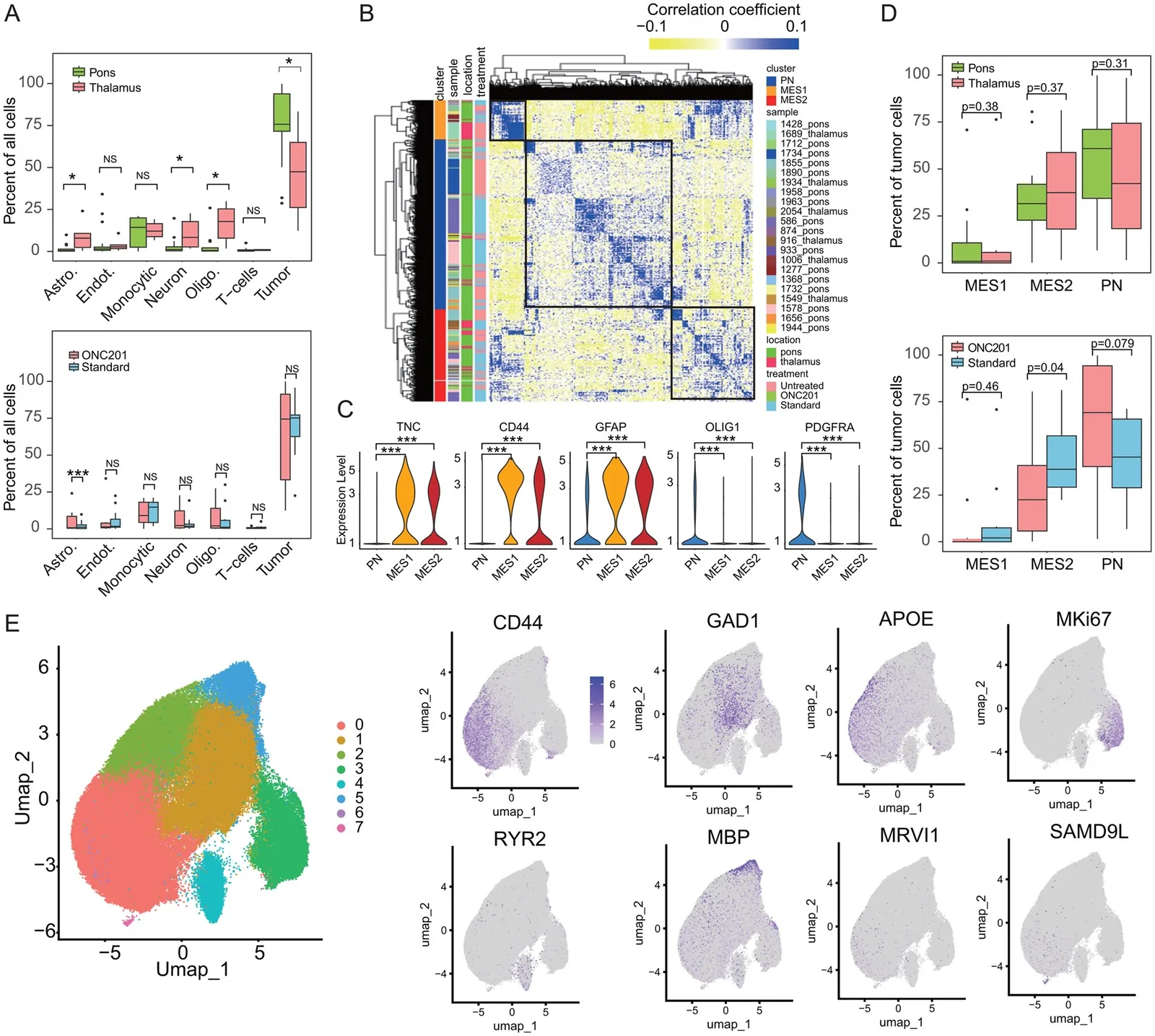
Imipridone treatment attenuates mesenchymal transition. (A) Comparison of cellular composition between tumor location (top) and therapeutic treatment (bottom). (B) Hierarchical clustering of DMG snRNA-seq identified proneural and mesenchymal clusters. (C) Violin plots of proneural and mesenchymal hallmark genes across clusters. (D) Comparisons of tumor composition based on identified clusters, contrasted by anatomical location (top) and treatment (bottom). (E) Unbiased clustering of snRNA-seq of neoplastic cells.

**Figure 3.**
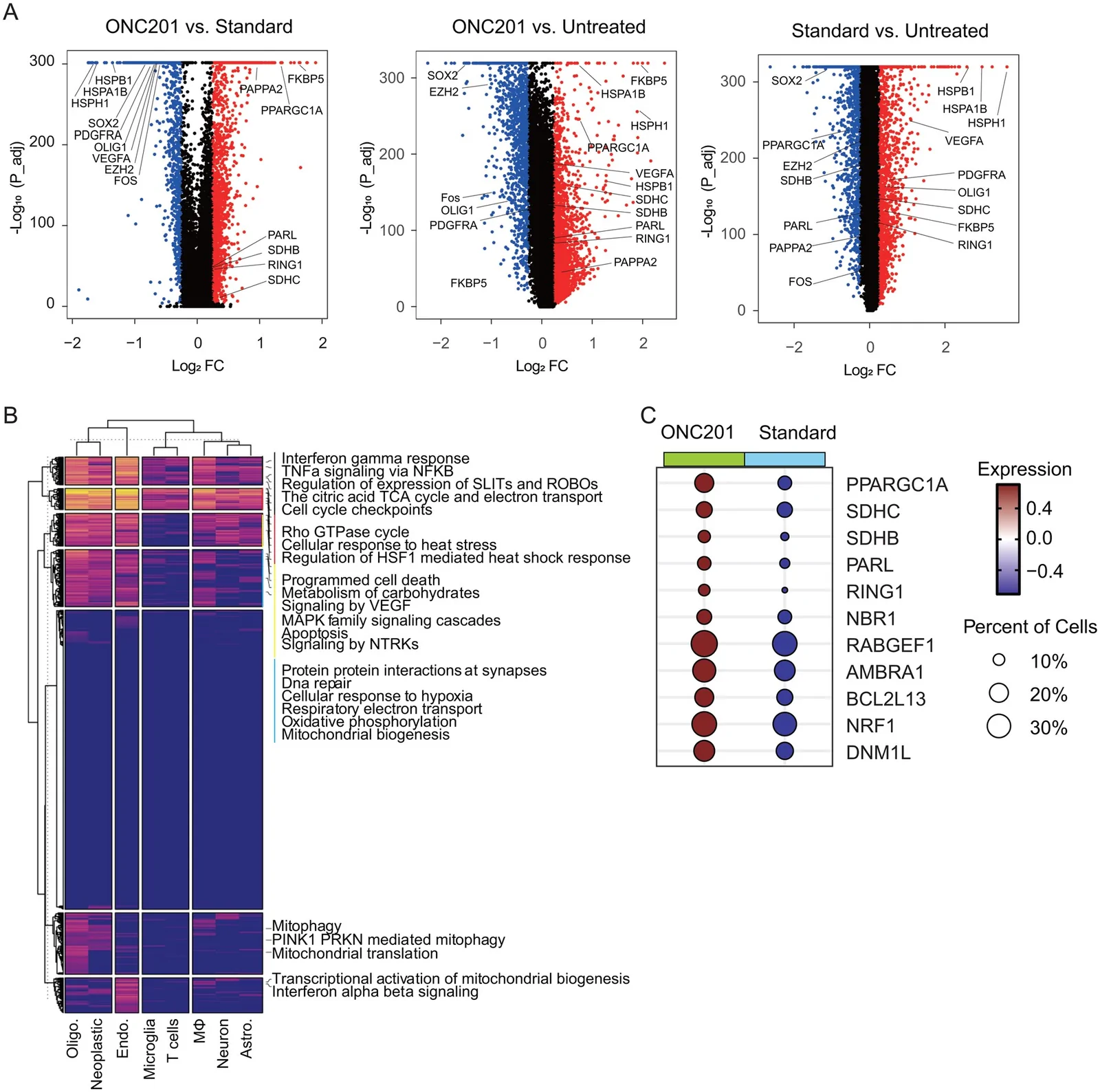
Differential expression analysis. (A) Differential expression tests between imipridone-treated, standard-care-treated, and untreated specimens. (B) Hierarchical clustering of differentially expressed genes by cell type, with over-represented gene-ontology terms annotated. (C) Expression of mitochondrial biogenesis genes.

The percentages of non-malignant cell types observed were largely unchanged between imipridone- and standard-care-treated cases, except for astrocytes (Figure 2A). However, when comparing thalamic tumors to pontine tumors, we found a significant increase in tumor-associated neuroglia and a decrease in tumor purity. We then performed a hierarchical clustering of neoplastic cells (Methods). This identified 3 transcriptional clusters (Figure 2B), expressing proneural (PN; Figure 2C, Figure S2D) and mesenchymal (MES1, MES2; Figure 2C, Figure S2D) markers, respectively.^17^^,^^32^ A differential composition test identified a decrease in mesenchymal cells in imipridone-treated vs. standard-care-treated cases, although no difference was observed in mesenchymal signatures between pontine and thalamic tumors (Figure 2D). An unbiased clustering (Methods) identified mesenchymal, progenitor, cycling, and other gene expression signatures (Figure 2E; Table S2) as well as heterogeneous RAS/MAPK pathway activation (Figure S2E). A differential expression test confirmed a decrease in mesenchymal gene expression between imipridone and standard-care-treated neoplastic cells (Figure 3A, Figure S3; Tables S3-S5). Imipridone-treated neoplastic cells upregulated TRAIL signaling, consistent with earlier preclinical studies.^35^ Radiation treatment induces a cellular stress response, including the expression of heat-shock proteins (HSPs), which promote tumor survival and therapy resistance.^36^^,^^37^ A large number of HSPs and associated genes were down-regulated in imipridone-treated cases compared to standard-care-treated specimens (Figure S3B-D). Intriguingly, imipridone-treated cases upregulated genes associated with mitochondrial biogenesis (Figure 3). In particular, *PPARGC1A,* a master-regulator of mitochondrial biogenesis,^38^ was significantly upregulated (Figure 3, Figure S3A; Table S3) in imipridone-treated samples compared to standard-care and untreated samples. Moreover, many targets of PPARGC1A and other genes associated with mitochondrial biogenesis and turnover were upregulated in imipridone-treated cases (eg *SDHB/C*, *PARL*, *RING1*, *NBR1*). This indicates that mitochondrial biogenesis may be a resistance mechanism to imipridone therapy.

**Figure 4.**
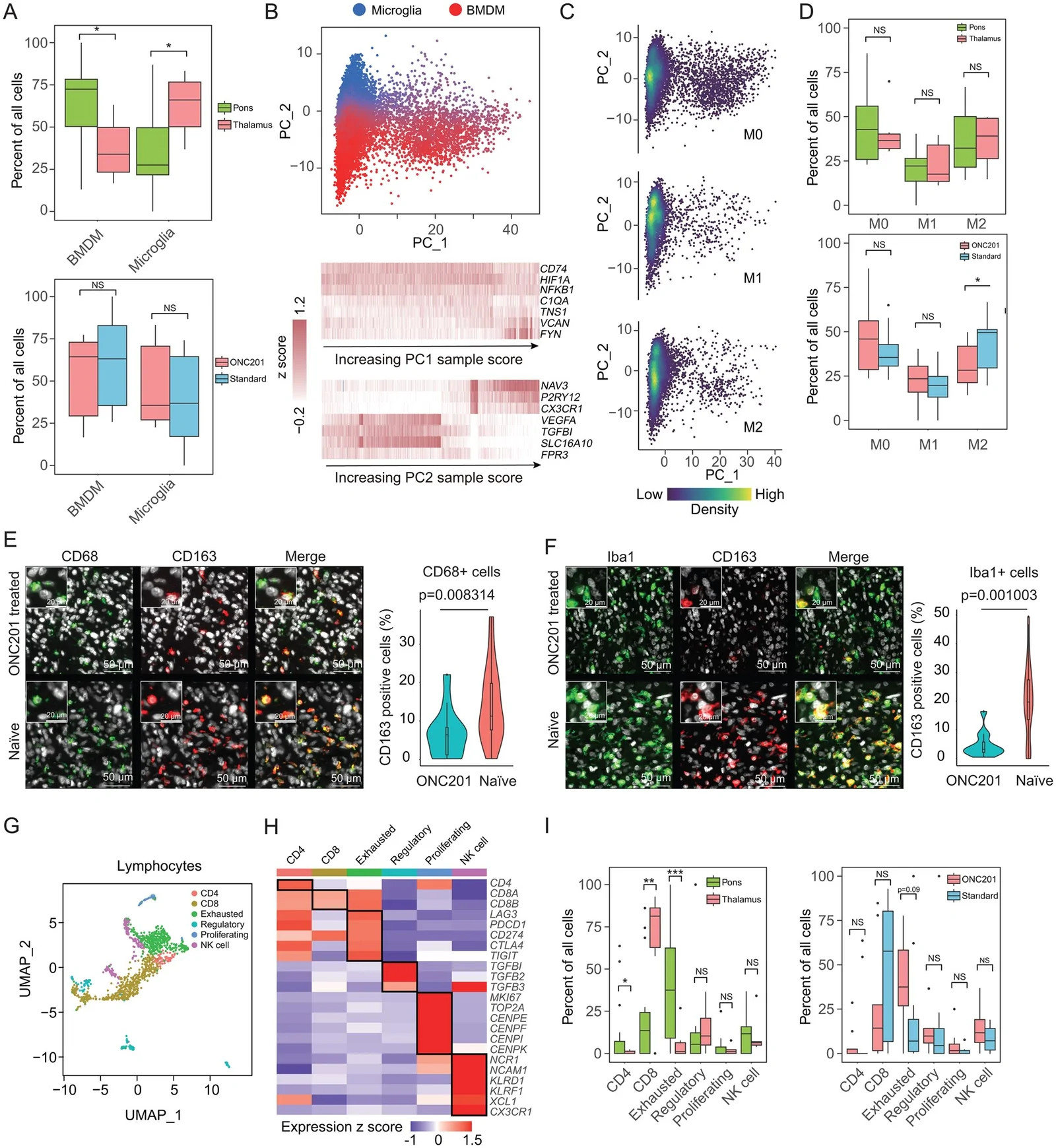
Analysis of expression in immune cells. (A) Percentages of myeloid cells depending on tumor location (top) or therapeutic treatment (bottom). (B) Top, PCA plot of myeloid cells. Bottom, gene expression sorted by principal component loading. (C) Density plot of M0, M1, and M2 monocytic lineage cells in PCA space. (D) Percentages of M0, M1, and M2 monocytic lineage cells by tumor location (top) or therapeutic treatment (bottom). (E-F) Representative IF images of the M2 activation marker CD163 in CD68+ and Iba1+ myeloid populations in imipridone-treated and naïve human DMGs (left). Quantification of CD163+ cells in CD68+ and Iba1+ myeloid cells in *N* = 48 DMGs (right). Significance is assessed via a Wilcoxon test populations across samples. (G) tSNE plot of lymphoid cells. (H) Heatmap of gene expression for hallmarks of canonical activation states. (I) Percentages of exhausted, regulatory, and proliferating lymphoid cells depending on tumor location (left) and therapeutic treatment (right).

We then classified monocytic lineage cells by ontogeny and activation status (Figure 4A-D, Figure S4). We found that monocytic lineage cells from DMGs were primarily microglia and had fewer myeloid cell infiltrates derived from circulation than adult glioblastomas (GBMs)^17^ (Figure 4A). Moreover, there were significantly higher levels of microglia than peripheral myeloid cells in thalamic tumors compared to those from the pons (Figure 4A). While we did not observe significant differences in ontogeny between treatment conditions, we did find significant differences in activation status. In particular, imipridone-treated cases showed significant decreases in the percentages of cells expressing M2 markers compared to standard-care cases (Figure 4D). To validate this finding, we subjected an orthogonal cohort of human DMG autopsy samples (*N* = 46 patients, 2-4 different brain locations per patient, 12 ONC201-treated, 34 ONC201-naïve) to immunofluorescence (IF) staining for protein markers of myeloid identity (CD68, Iba1) and M2 activation (CD163; Methods). We found that the percentages of M2-activated myeloid populations were significantly decreased in imipridone-treated cases (Figure 4E and F). We then classified T cells by activation status (Figure 4G-I). Similar to adult GBMs,^17^ we found exceedingly few proliferative T cells that expressed markers of cytotoxic activity. Rather, most cells expressed markers of exhaustion, and there was no significant difference in activation across treatment conditions (Figure 4G). Lastly, HSPs were broadly downregulated in immune cells (as observed in neoplastic cells), as were negative regulators of antigen presentation (Tables S3-S5).

### Single-Nucleus ATAC Sequencing Shows That Imipridone-Treated Cases Have Globally Reduced Enhancer Activity Consistent With the Theoretical Imipridone Mechanism of Action

**Figure 5.**
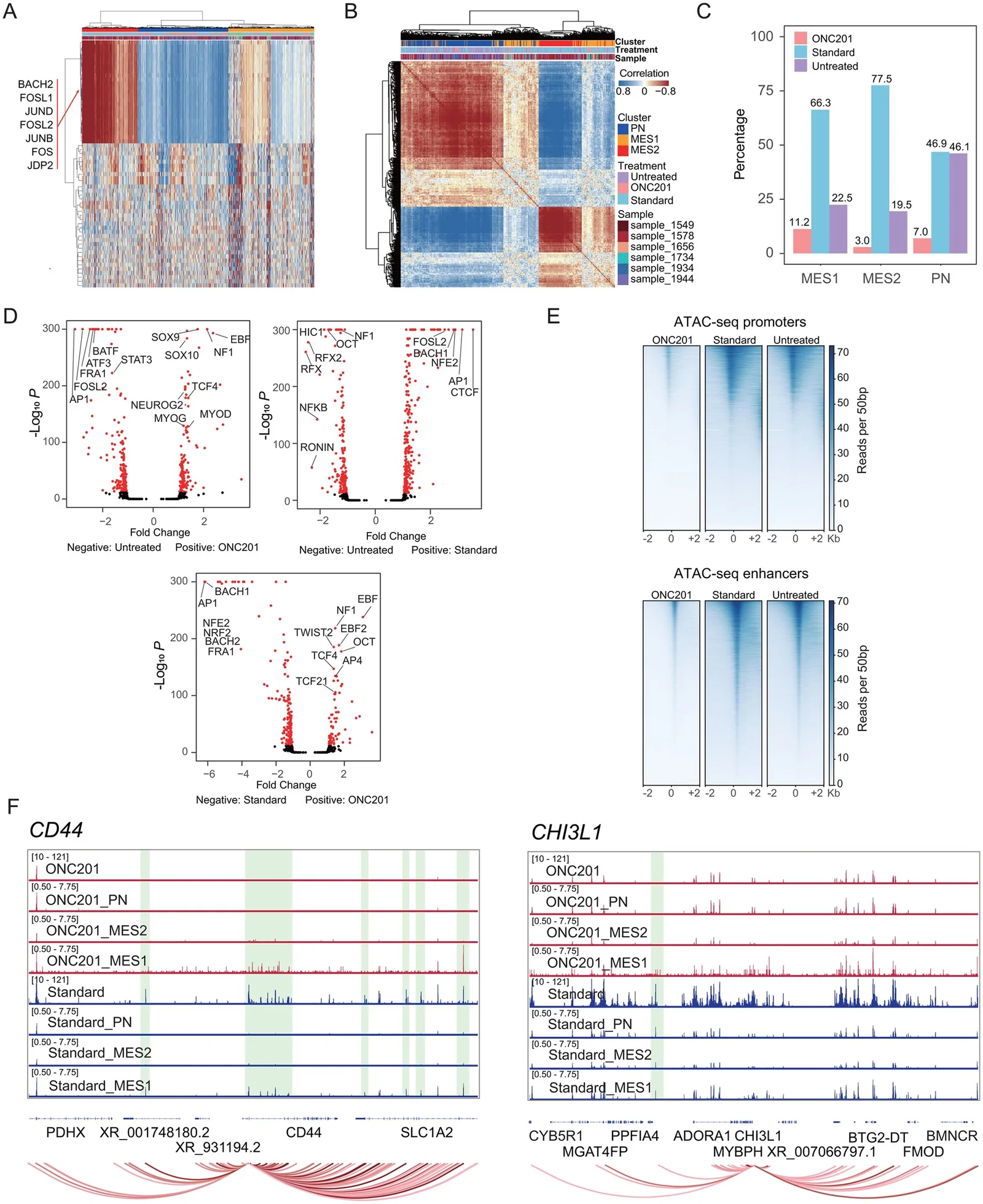
Analysis of snATAC-seq data. (A) Hierarchical clustering of cells based on transcription-factor motif frequencies, represented as standard scores relative to a data-driven background. (B) Hierarchical clustering of inter-cell correlations in transcription-factor motif frequencies. (C) The percentages of mesenchymal and proneural clusters by treatment condition. (D) Differential motif frequency tests between imipridone-treated, standard-care-treated, and untreated specimens. (E) Global quantification of snATAC-seq reads mapping to promoters (top) and enhancers (bottom). (F) Browser plots of enhancers whose activity is specific to standard-care vs. imipridone therapy and which correlate with expression of the associated gene.

H3K27M mutations have been shown to globally upregulate gene expression by decondensing chromatin and decreasing histone methylation at gene enhancers. In model systems, imipridone treatment has been reported to antagonize this phenomenon, globally decreasing gene expression by muting enhancer activity.^9^ To test if enhancer muting would be observed clinically, we interrogated our matched snATAC-seq data (Figure 5A-C; Table S1). Following quality control and preprocessing, we identified neoplastic cells based on expressed mutations (Figure S5A) and classified non-malignant cell types based on gene-body activity (Figure S5B; Methods). We used chromVar^21^ to identify TF motifs present in the snATAC-seq sequenced reads and to quantify their frequencies relative to a data-driven background estimate. We then clustered cells by TF motif frequencies and identified 3 clusters of cells stratified by the representation of PN (eg ATOH1, OLIG2, NEUROD1/G2) and MES (eg JUN/JUNA/JUNB, FOS, BACH1/2) TF motifs (Figure 5A and B, Figure S5C). Confirming our snRNA-seq analysis, we found a decrease in the percentage of MES cells in imipridone-treated samples (Figure 5C). Differential motif-frequency tests (Figure 5D, Figure S5C; Methods) confirmed enrichments for PN and MES TF activities in imipridone-treated vs. standard-care-treated specimens. We then projected snRNA-seq and snATAC-seq data into a common latent space to associate gene-expression profiles with snATAC-seq data, and thereby correlate gene expression with open chromatin to infer cis-regulatory enhancers (Methods). We found a global decrease in enhancer activity in the neoplastic cells derived from imipridone-treated cases (Figure 5E), consistent with the predictions of recent preclinical models.^9^ In particular, we found that the differential expression of PN and MES genes between treatment conditions (and other differentially expressed genes) could be explained by differential cis-regulatory enhancer activity (Figure 5F, Figure S5D). Taken together, these data indicate that, clinically, imipridone treatment globally mutes gene expression by down-regulating associated enhancers.

### Imipridone Treatment Reduces Immunosuppressive Paracrine Signaling With Increased Effect in Thalamic Tumors

**Figure 6.**
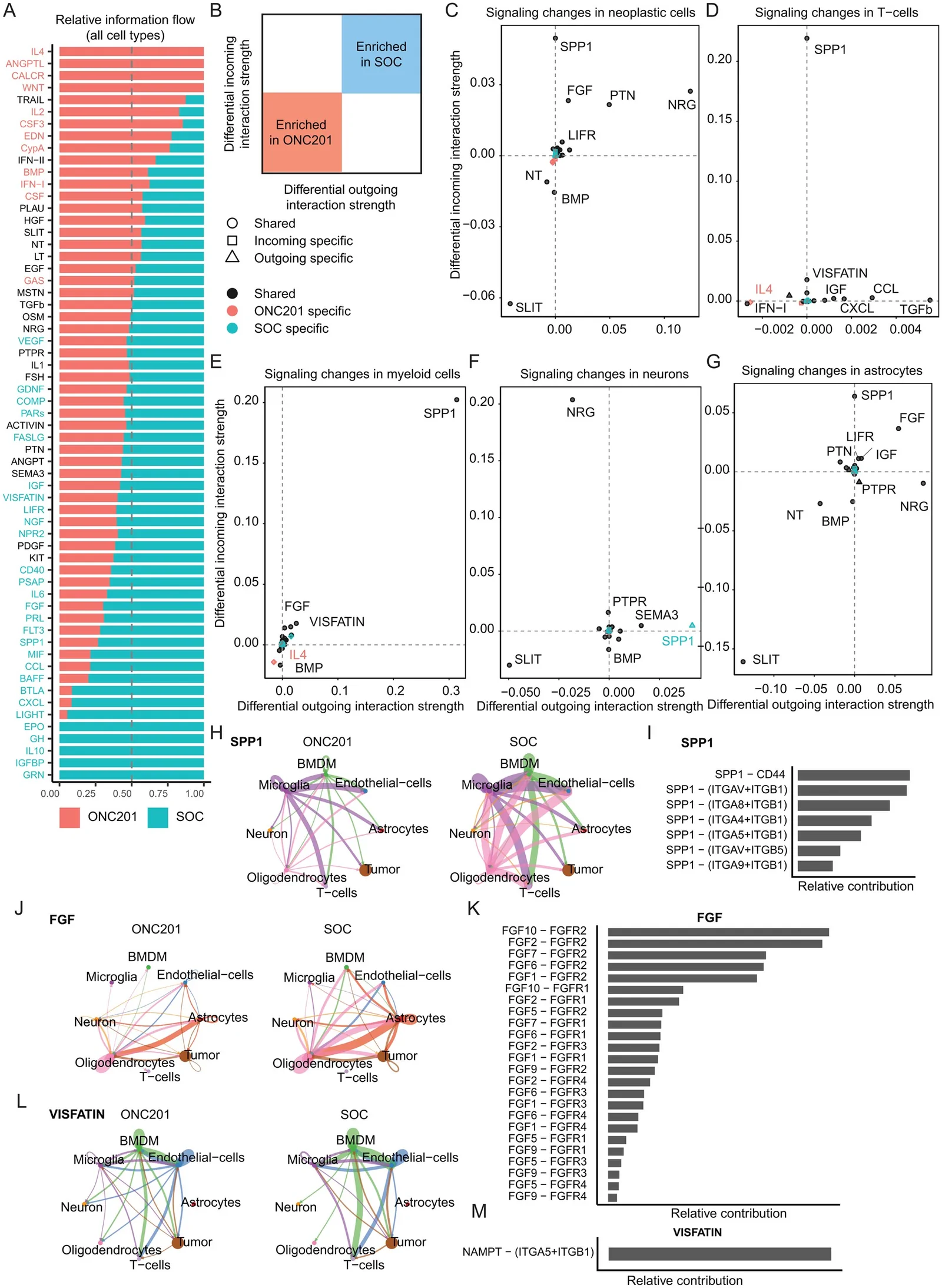
Paracrine signaling analysis. (A) Relative information flow metric, identifying differentially active paracrine signaling pathways between imipridone and standard-care treated specimens. (B) A schematic illustrating how to interpret the dot plots in panels C-G. (C-G) Dot plots indicating paracrine signaling pathways that are differentially active between imipridone and standard-care-treated specimens, by cell type. (H-M) Network diagrams of paracrine signaling networks that are differentially active and their associated relative contribution of specific ligand-receptor pairs in those pathways.

We then performed an analysis of global paracrine signaling networks from snRNA-seq data via CellChat.^24^ Consistent with the decrease in M2 immunosuppressive myeloid cells we observed in imipridone-treated cases (Figure 4), we observed downregulation of IL10, CXCL12, SPP1, and other pathways previously implicated in local immunosuppression and myeloid cell chemotaxis (Figure 6, Figure S6). Intriguingly, among cases treated with standard therapy alone, thalamic tumors showed greater diversity of paracrine signals. This differential activity was largely attributable to neuroglia and microglia and consistent with the differential abundance of these cell types observed in thalamic tumors. Imipridone-treated thalamic cases specifically showed a marked reduction of *SPP1*, *PTN*, *IL10*, and other paracrine signals that support glioma growth and promote immunosuppression.

### Imipridone Treatment Induces PPARGC1A Expression and Mitochondrial Biogenesis in Treatment-Resistant Cells

**Figure 7.**
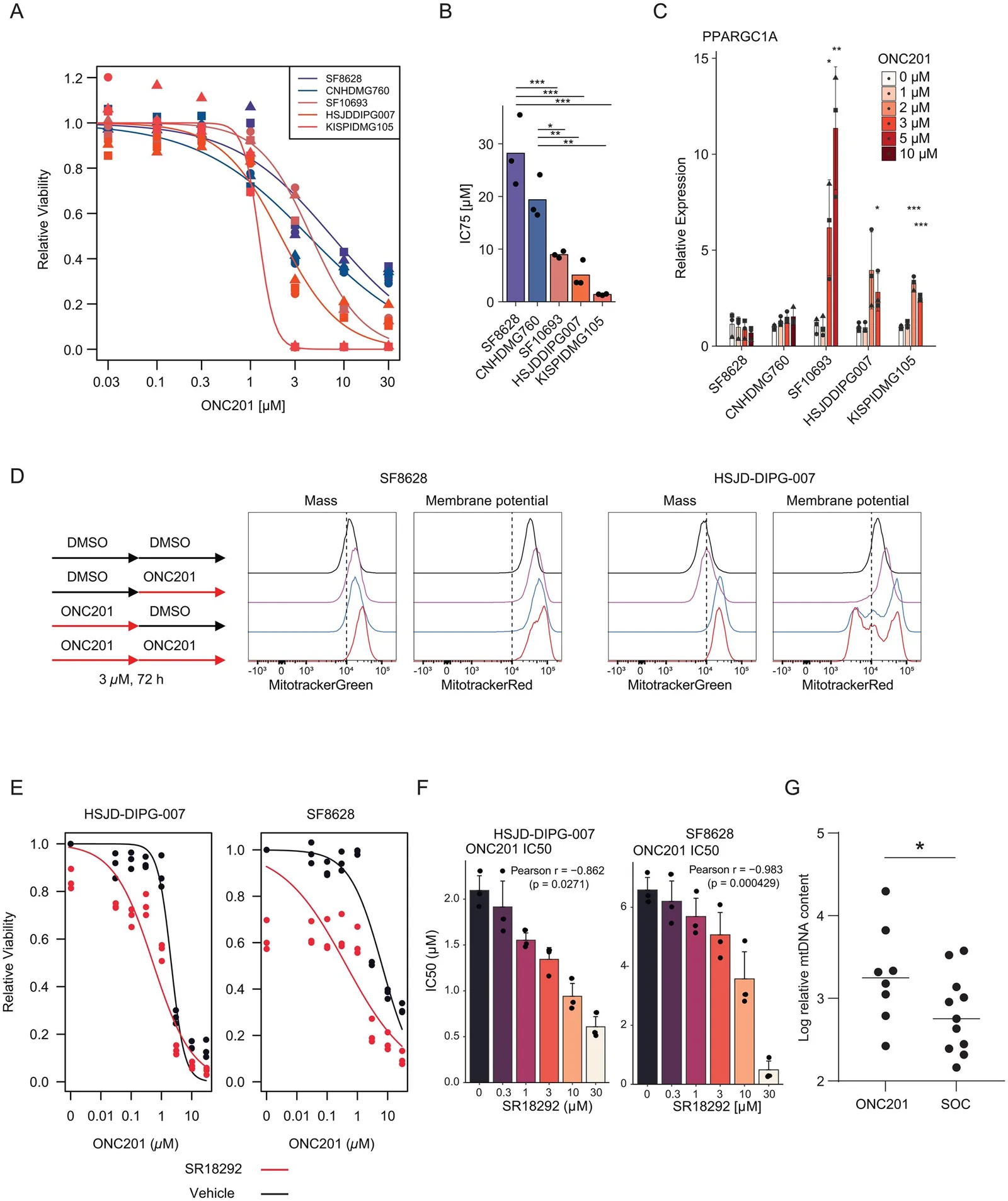
Imipridone treatment increases mitochondrial biogenesis through the induction of PPARGC1A. (A) ONC201 dose-response curves for *N* = 5 DMG cell lines, from 3 independent experiments. (B) IC75 values from panel A. Significance was assessed via one-way ANOVA followed by Tukey’s test; *: *P* < .05, **: *P* < .01, ***: *P* < .001. (C) QPCR of *PPARGC1A* across cell lines, from 3 independent experiments. Significance was assessed via an unpaired t test; *: *P* < .05, **: *P* < .01, ***: *P* < .001. (D) Quantification of mitochondrial mass and membrane potential of imipridone-treated DMG cells and DMSO controls by cytometric analysis of MitoTracker Green and Deep Red, respectively. DMG cells were treated with DMSO or 3 µM ONC201 for 72 hours and then washed. Subsequently, cultures were subjected to an additional round of ONC201 or DMSO treatment for 72 hours. Data were pooled from 3 independent experiments. (E) In vitro survival curves comparing the ONC201 dose response between PPARGC1A-inhibitor (30 µM SR18292) and control conditions. (F) IC50 plotted versus dosage of PPARGC1A inhibitor SR18292. (G) Mitochondrial DNA content was compared between ONC201-treated and ONC201-naïve postmortem human DMGs. * - One-sided Mann-Whitney U-test < 0.05.

We treated human primary DMG cell lines (*N* = 5) with the imipridone ONC201 and observed varying degrees of sensitivity (Figure 7A and B). We found that ONC201-sensitive lines upregulated *PPARGC1A* in response to ONC201 treatment, but that resistant lines did not (Figure 7C). We compared mitochondrial density and membrane potential between a sensitive (DIPG-007) and a resistant (SF8628) line. We found that both metrics were significantly higher in the untreated resistant line SF8628 (Figure 7D). However, in both cell lines, we found that 72h ONC201 treatment led to increased mitochondrial density and membrane potential, and that this change was durable after a 72-hour washout period (Figure 7D). Prolonged exposure to ONC201 (144 h) induced a bimodal distribution of mitochondria with high and low membrane potentials, indicating that imipridone therapy may select mitochondria with high membrane potentials.

We observed increased expression of *PPARGC1A* and other genes associated with mitochondrial biogenesis/turnover in imipridone-treated cases (Figure 3A and B; Table S3). Therefore, we combined a PPARGC1A inhibitor, SR18292,^39^ with ONC201. We found that SR18292 treatment significantly increased sensitivity to ONC201 (Figure 7E and F) and synergized with ONC201 (Figure S7). The resistant cell line SF8626 showed a greater increase in sensitivity to ONC201 upon inhibitor treatment. We then sought to determine if imipridone-treated cases demonstrated increased mitochondrial density post-therapy. To that end, we applied PCR-based quantification of mitochondrial DNA copy number^40^ to *N* = 8 ONC201-treated and *N* = 11 ONC201-naïve post-mortem human DMGs (Table S1). We found a significant increase in mitochondrial DNA in imipridone-treated residual disease (Figure 7G).

## Discussion

We conducted a multi-modal genomics analysis of postmortem specimens derived from imipridone-treated and imipridone-naïve patients. We extended observations on imipridone mechanisms from prior preclinical studies and identified mitochondrial biogenesis as a resistance mechanism and a target for adjuvant therapy development. We found that imipridone treatment significantly altered tumor composition. Mesenchymal transition is a hallmark of therapy resistance in glioma and is driven by Activator-protein 1 (AP1) signaling.^5^^,^^17^^,^^41^^,^^42^ Our data indicate that imipridone treatment results in regression of the mesenchymal signature and downregulation of the gene components of the AP1 complex (Figures 2, 3A, and 5A-D).

Mesenchymal glioma cells co-localize with myeloid-derived suppressive cells both across patients and spatially within distinct regions of individual tumors.^17^^,^^32^ These populations appear to engage in a mutually reinforcing interaction, in which myeloid-derived cytokines can promote mesenchymal transition.^17^^,^^32^^,^^43-45^ We found that immunosuppressive M2 myeloid-derived cells were significantly decreased in imipridone-treated DMGs (Figure 4A-F). These findings support the potential of imipridones as adjuvants for emerging immunotherapies. Indeed, a recent study has shown synergistic efficacy of ONC201 in combination with B7H3 CAR-T therapy in DMG preclinical models.^46^

Prior preclinical studies demonstrated that imipridone treatment globally downregulates gene-enhancer activity through increased histone methylation.^9^ Aberrant activation of gene enhancers is a hallmark of DMG, and the evidence supports that this results from inefficient chromatin compaction at enhancers due to H3K27M mutations.^9^ We found that this phenomenon of global enhancer suppression via imipridone therapy occurs clinically (Figure 5E). Most downregulated genes in imipridone-treated cases could be explained by differential cis-regulatory enhancer activity (Figure 5F, Figure S5D). On the other hand, genes involved in the mitochondrial respiratory chain, mitophagy, mitochondrial damage quality control, and mitogenesis were specifically upregulated in imipridone-treated tumors (Figure 3). In an orthogonal cohort, mitochondrial density was increased in imipridone-treated cases (Figure 7G). Our drug-challenge experiments demonstrated a significant increase in mitochondrial density following imipridone treatment (Figure 7D) and activation of PPARGC1A, a master regulator of mitochondrial biogenesis (Figure 7C). Small-molecule inhibition of PPARGC1A enhanced sensitivity to imipridone treatment, even in a previously resistant line (Figure 7E and F). Taken together, these data suggest that imipridone treatment drives a compensatory mitochondrial program rather than a purely cytotoxic response. The coordinated upregulation of pathways linked to the respiratory chain, mitophagy, mitochondrial quality control, and mitochondrial biogenesis is consistent with tumors’ preservation of bioenergetic capacity and their ability to limit damage under drug-induced stress. In this context, the observed increase in mitochondrial density across cohorts can be interpreted as an adaptive remodeling of mitochondrial content, potentially enabling persistence despite therapy.

A key implication is the apparent centrality of PPARGC1A-mediated mitochondrial biogenesis in this adaptation. PPARGC1A is well-positioned to integrate stress cues and orchestrate increased mitochondrial mass, and its induction following imipridone exposure supports a model in which mitochondrial biogenesis functions as a survival mechanism. The finding that pharmacologic inhibition of PPARGC1A enhances imipridone sensitivity, even in a previously resistant line, further strengthens the argument that this pathway is not merely a bystander signature but a functional determinant of response.

More broadly, these observations point to mitochondrial biogenesis as a rational co-target to improve imipridone efficacy. If mitochondrial expansion results in buffering metabolic and oxidative stress imposed by imipridones, then suppressing this adaptive response may lower the threshold for apoptosis or growth arrest and prevent or delay resistance. This framework motivates combination strategies that pair imipridones with inhibitors of PPARGC1A signaling or downstream mitochondrial biogenesis machinery and suggests that mitochondrial biogenesis markers may have value as pharmacodynamic readouts and as predictors of benefit from such combinations.

Limitations of this study include its largely correlative nature. This is partially offset by the use of rare clinical specimens, which avoid some of the artifacts inherent to preclinical models, as well as by the value of these multi-omic data as a resource for the field. Future studies using models of acquired resistance, such as long-term imipridone exposure in patient-derived xenografts or organoids, will be important to further establish causality. In addition, because postmortem specimens were used, mitochondrial programs could potentially be influenced by the postmortem interval. However, we did not observe a correlation between postmortem interval and standard snRNA-seq quality metrics or mitochondrial biogenesis-related gene or signature scores in our cohort (Figure S1G). Moreover, the principal mitochondrial biogenesis findings were supported in an orthogonal cohort (Figure 7G) and in vitro (Figure 7C-F), arguing that they are unlikely to be attributable to post-mortem artifact.

Imipridone therapy has the potential to alter the treatment landscape for DMG, but several barriers remain before broad clinical adoption is feasible. In this context, our findings add to a growing body of work by confirming key observations from preclinical models in human tumors and by sharpening the rationale for combination approaches that may enhance efficacy and delay resistance, including strategies that disrupt adaptive mitochondrial programs induced by imipridones.

## Supplementary Material

noag119_Supplementary_Data
