# Supplementary material for: Single-nucleus profiling of postmortem diffuse midline gliomas identifies mitochondrial biogenesis as a resistance mechanism to imipridone therapy
Source: Neuro Oncol. 2026 May 24;28(9):2248–62. doi: 10.1093/neuonc/noag119 (PMC13550678; doi:10.1093/neuonc/noag119)
Supplement: Supplementary file 1 [file noag119_supplementary_data.zip › Supplementary Table and Figure Legends.docx]

**Supplementary table legends**

Table S1: A summary of patient samples used in this study.

Table S2: Cluster-specific genes from an unbiased clustering of snRNA-seq of neoplastic cells.

Table S3: Differential expression between ONC201 and standard-care treated specimens.

Table S4: Differential expression between ONC201-treated specimens and an untreated case.

Table S5: Differential expression between standard-care-treated specimens and an untreated case.

**Supplementary figure legends**

*Figure S1:* (A) Feature counts by sample. (B-F) UMAP representation of snRNA-seq by treatment, age, location, sample ID, and gender, respectively. (G) Correlation between the number of features detected in snRNA-seq and the time to tissue processing post mortem.

*Figure S2:* (A-C) Copy-number variant analysis by treatment condition. Significance was assessed via a two-sided t-test, *: p<0.05. (D) Expression of mesenchymal and proneural hallmark genes in the expression clusters of Figure 2. (E) Expression of RAS/MAPK pathway genes across samples.

*Figure S3:* (A) Heatmap of the relative expression of the mitochondrial biogenesis gene set across cases. (B) Violin plots for specific heat-shock proteins between treatment conditions, by cell type. All genes listed are statistically significant in the associated differential expression test with q<0.05 (Tables 2-4). (C-D) Over-represented gene ontology terms in the differential expression tests of Figure 3.

*Figure S4:* UMAP and tSNE plots of snRNA-seq of monocytic lineage cells (A-F) and lymphoid cells (G-K), by treatment condition, location, gender, cell-type, age, and sample.

*Figure S5:* (A) tSNE plot of the snATAC-seq data with clonal CNVs annotated. (B) Differential composition analysis by tumor location (left) and treatment (right). Significance was assessed via a two-sided t-test; *: p < 0.05, **: p < 0.01, ***: p < 0.001. (C) Cluster-specific over-represented motifs. (D) Browser plots of enhancers whose activity is specific to standard-care vs. imipridone therapy and which correlate with expression of the associated gene.

*Figure S6:* Network diagrams of paracrine signaling networks that are differentially active and their associated relative contribution of specific ligand-receptor pairs in those pathways.

*Figure S7:* Dose response and Loewe synergy scores for ONC201 vs PPARGC1A inhibitor SR18292 in SF8628 (A) and DIPG 007 (B) cell lines.
